# Some Conclusions in Regard to General Paresis, with the Report of a Case under Observation

**Published:** 1873-07

**Authors:** Horatio R. Bigelow

**Affiliations:** Boston, Mass.


*Some Conclusions in Regard to General Paresis, with the Report of a Case under Observation.* By HORATIO R. BIGELOW, Boston, Mass.

A case of general paresis now under treatment, although only in the first of the three stages described by Calmeil, suggests a few conclusions in regard to some of the characteristic nervous phenomena which form a prominent symptom in the development of the disease.

*First of the Case.*—The patient is a man fifty years old, tall and stout; the forehead is very narrow transversely, depressed in the region of the spheno-frontal articulation and at the cranial and vertex; the face, when at rest, is entirely devoid of expression, the integumental folds are obliterated; the chin is corrugated from contraction of the levator-menti muscle; the complexion is sallow; the eyes are sleepy and dull, the pupil of the right being larger than that of the left, and dilating irregularly; there is an air of perfect placidity and great self-importance about the patient's demeanor; the appetite is almost voracious, at the same time that it is capricious; there is, also, occasional regurgitation of the food.

*Local Alterations.*—Muscles of tongue affected; there is hesitancy of utterance, inability to pronounce the labials correctly, a slurring, guttural manner of speech, but with no disposition to garrulousness, the patient recognizing his own defects; while giving utterance to certain words the head is thrown slightly upward, and the lower lip twitches spasmodically, conveying a peculiar motion to the chin; the tongue alternately contracts and relaxes when protruded.

*Motor Functions.*—Some of the local phenomena might point to a more advanced stage of the disease, were it not for the fact that the motor functions of the extremities are not perceptibly implicated; the patient's walk is a language of its own, it conveys the entire sense of egotistical importance, that entire indifference to other mortals which is so characteristic of the mental condition of the patient; the foot, but slightly raised from the ground, is advanced slowly, with but little flexion of the leg, and planted flat on the ground with a determined air.

*Mental Condition.*—There is unvarying contentment of mind, buoyancy of spirit, and unclouded hope; to an interrogation as to the state of his health, he would make reply, "First rate; never better," etc.; he is fond of discoursing upon the extent of his business, his charming residence, and his family connections; he is contemplating a tour on the continent, with his family, to extend over a period of many months; his memory of past events is perfect, but he cannot remember the substance of what he has read five minutes previously; he has developed a decided tendency to kleptomania of late, using much ingenuity in concealing his depredations; he has great elation of ideas, and sees everything *couleur de rose.*

*Ophthalmoscopic Signs.*—Congestion (slight) of the disk.

I am aware that this case presents no features hitherto unknown to the profession, but it is an excuse for dwelling somewhat upon the value of the ophthalmoscope in the diagnosis of cerebral diseases, and for advancing a few theories in regard to the mental implications.

I am indebted to my friend Dr. R. A. Vance, an eminent practitioner of New York City, for the valuable data in relation to the ophthalmoscopic signs in general paresis.

"In every case of general paralysis that has fallen under my observation, the ophthalmoscope has revealed morbid changes of a vascular, neuritic, or atrophic character. In thirty-one cases of which I have notes of the intra-ocular appearances at the time I first examined them with the ophthalmoscope, eleven presented evidences of atrophy of the disk and surrounding parts of the retina, thirteen of neuro-retinitis, and seven of congestion of the disk and retina. Those cases in which neuritic and atrophic changes were marked were of long standing, while those in which vascular derangement alone was present were in the early stages of the disease. In three out of seven cases characterized by congestion of the intra-ocular structures, repeated ophthalmoscopic observations demonstrated the subsequent development of neuro-retinitis, which finally terminated in atrophy of the intra-ocular portion of the optic nerve. The rapidity with which the neuritic and atrophic changes succeed the congestive appearances bears no relation to the general progress of the intra-cranial disease, but seems to depend upon local causes which, as yet, have not been determined."

The "elation of the ideas" is due to a vicious action of the vesicular neurine of the ideational centres, rather than to an exaltation of the faculties of the mind. The molecular condition representing imagination undergoes a specific, minute change, by which its harmonious action with the centres of judgment becomes disrupted, and commonplace expression results. One of the first appreciable mental changes of general paresis consists in this perverted imagination, this intellectual feebleness; and from the consideration of these symptoms we are led to a probable location of the universal lesion. There is in this disease a very manifest want of emotional control. Now, as emotion depends upon the sensibility of the vesicular neurine to ideas, and as the idea depends upon the impression made upon the supreme centres,* it follows that any molecular change of this latter will affect all the mental organization, since we believe that the human mind is the perfected harmonious *force*, generated by the ideational centres, and that this force will vary in intensity according as it is evolved by a more or less intricate arrangement of the cerebral convolutions, and from a small or large number of cells. The emotional aberration depends directly upon a degeneration of will, which we should expect to be the case, as no such abstraction of *the will*, apart from its mental relationship, has a recognized existence. That memory preserves its integrity to an advanced period in paresis may be due to the fact that the centres in which ideas are registered are the last to yield to the vicious action, or that the residual force of the previous normal condition thus stored up discharges with fidelity its routine of the past, without having sufficient vital organization to retain impressions of the present. Corroborating instances of this species of conservatism are by no means rare in the life of private practitioners, being frequently met with in the course of certain febrile and cerebral diseases. In every organic element of the body there is this registration of ideas, and the impression once made is indestructible; but as the integrity of action depends upon the harmonious assimilation of philosophical ideas, the retentive power may be perverted or obscured by an abnormal condition of the ideational centres. From the relation and assimilation of ideas emanate imagination, hence a vivid imagination would result from some molecular change in the centres generating the idea, while an unhealthy imagination would depend upon diseased action of those centres. Whether the paralysis precedes the mental degeneration, or is preceded by it, must, at present, be considered as *sub judice;* but I am inclined to believe that, in the great majority of cases, want of motor co-ordination is secondary to the nervous lesion. This supposition is based upon a knowledge of the intimate and dependent relationship of the sensory-motor and higher nervous centres. A disease of one centre, by a process of vicarious emigration, may convey its contaminating influence to a very remote cell, whose functions in the processes of life and thought may be of an entirely different nature, thus developing a complex irregularity out of the original simple lesion. Who shall limit the extent of power in the higher centres? or who can measure the dependence of the physical upon the psychical? The minutest polar change in the molecular arrangement of the vesicular neurine of these supreme centres is felt, sometimes inappreciably, throughout the human

* Maudsley's "Body and Mind."

organism. A prick of a pin conveys to a special department its sense of pain, and immediately a reflex action ensues in the member thus abused; but if disease interrupt the action of this nervous centre, anæsthesia of the parts supplied by it necessarily results, so soon as the primary residual force shall have spent itself. In those cases of general paresis in which it is asserted that the paralysis manifested itself primarily, it is more than probable that the mental lesion did exist, but manifested itself by such slight external symptoms as to have been overlooked.—*Canada Med. and Surg. Journal.*

